# Global Sensitivity Analysis of Metabolic Models for Phosphorus Accumulating Organisms in Enhanced Biological Phosphorus Removal

**DOI:** 10.3389/fbioe.2019.00234

**Published:** 2019-10-04

**Authors:** Minh Nguyen Quang, Tim Rogers, Jan Hofman, Ana B. Lanham

**Affiliations:** ^1^Department of Chemical Engineering, Water Innovation and Research Centre, University of Bath, Bath, United Kingdom; ^2^Department of Mathematical Sciences, Centre for Networks and Collective Behaviour, University of Bath, Bath, United Kingdom

**Keywords:** global sensitivity analysis, Monte Carlo, enhanced biological phosphorus removal, phosphorus accumulating organism, metabolic model, EBPR, standard regression coefficients, sobol sensitivity analysis

## Abstract

The aim of this study was to identify, quantify and prioritize for the first time the sources of uncertainty in a mechanistic model describing the anaerobic-aerobic metabolism of phosphorus accumulating organisms (PAO) in enhanced biological phosphorus removal (EBPR) systems. These wastewater treatment systems play an important role in preventing eutrophication and metabolic models provide an advanced tool for improving their stability via system design, monitoring and prediction. To this end, a global sensitivity analysis was conducted using standard regression coefficients and Sobol sensitivity indices, taking into account the effect of 39 input parameters on 10 output variables. Input uncertainty was characterized with data in the literature and propagated to the output using the Monte Carlo method. The low degree of linearity between input parameters and model outputs showed that model simplification by linearization can be pursued only in very well defined circumstances. Differences between first and total-order sensitivity indices showed that variance in model predictions was due to interactions between combinations of inputs, as opposed to the direct effect of individual inputs. The major sources of uncertainty affecting the prediction of liquid phase concentrations, as well as intra-cellular glycogen and poly-phosphate was due to 64% of the input parameters. In contrast, the contribution to variance in intra-cellular PHA constituents was uniformly distributed among all inputs. In addition to the intra-cellular biomass constituents, notably PHB, PH_2_MV and glycogen, uncertainty with respect to input parameters directly related to anaerobic propionate uptake, aerobic poly-phosphate formation, glycogen formation and temperature contributed most to the variance of all model outputs. Based on the distribution of total-order sensitivities, characterization of the influent stream and intra-cellular fractions of PHA can be expected to significantly improve model reliability. The variance of EBPR metabolic model predictions was quantified. The means to account for this variance, with respect to each quantity of interest, given knowledge of the corresponding input uncertainties, was prescribed. On this basis, possible avenues and pre-requisite requirements to simplify EBPR metabolic models for PAO, both structurally via linearization, as well as by reduction of the number of non-influential variables were outlined.

## 1. Introduction

Enhanced biological phosphorus removal (EBPR), a variation of conventional Activated Sludge (AS), is a widely employed technology to remove phosphorus from wastewater. This prevents eutrophication in waterways due to the excess of nutrients (Metcalf and Eddy, 2003; Oehmen et al., 2007). By engineering alternating anaerobic and aerobic conditions, the resultant community of phosphorus accumulating organisms (PAO) assimilates phosphorus from the bulk liquid phase by intra-cellular accumulation as poly-phosphate (poly-P). Under stable operation, EBPR installations have been demonstrated to achieve very high phosphorus removal efficiencies, with effluent phosphate (PO_4_) concentrations as low as 0.5 mg/l (Lopez-Vazquez et al., 2008a).

However, EBPR is known to suffer from operational instabilities from causes which are not completely understood. The deterioration of phosphorus removal performance is often attributed to the proliferation of glycogen accumulating organisms (GAO), which do not directly contribute to phosphorus removal yet compete with PAO for carbon sources, e.g., volatile fatty acids (VFA). In an effort to suppress their growth, different studies have investigated factors that influence the competition between these organisms, e.g., the carbon source (Pijuan et al., 2004; Oehmen et al., 2005b), pH (Filipe et al., 2001a,b,c), temperature (Whang and Park, 2006; Lopez-Vazquez et al., 2008b), the P/C ratio in the influent (Liu et al., 1997) and the aeration levels (Carvalheira et al., 2014a). Metabolic models, generally a more detailed approach than conventional activated sludge models (ASM), can be used to effectively describe their metabolisms and thereby consider the effect of these factors on the competition between PAO and GAO.

Metabolic models offer a way to integrate information from various sources within a common mathematical framework. They describe and predict the metabolism of key organisms relevant to EBPR on a mechanistic basis. The EBPR metabolic model stemmed mainly from the work of Smolders et al. (1994), describing the anaerobic metabolic pathways of PAO. It has since grown to include the aerobic (Smolders et al., 1995) and anoxic (Kuba et al., 1996) metabolisms of *Accumulibacter* PAO, as well as that of *Competibacter* and *Defluviicoccus*-related GAO (Filipe et al., 2001b; Zeng et al., 2003b; Oehmen et al., 2005b). These models have also expanded to include the effects of carbon source, temperature and pH (Oehmen et al., 2005a, 2006; Lopez-Vazquez et al., 2009). Interfaced with ASM, they have been successfully used to describe the performance of full-scale EBPR installations (Van Veldhuizen et al., 1999; Meijer et al., 2001). Furthermore, they offer a means with which to test hypotheses concerning microbial ecology and explore their population dynamics under multi-parametric settings, e.g., as conducted by Lopez-Vazquez et al. (2009) between PAO and GAO, as well as within their respective sub-groups, e.g., in Oehmen et al. (2010b) between *Accumulibacter* PAO clades I and II.

In recent years, the application of advanced molecular techniques has led to a more comprehensive understanding of both the metabolic activity and phylogenetic diversity (Oehmen et al., 2007), e.g., differing capacities of PAO clades for denitrification (Zeng et al., 2003a; Flowers et al., 2009; Oehmen et al., 2010b), utilization of the TCA cycle to supply reducing power for PHA formation (Lanham et al., 2014) or shift to glycolysis-driven VFA uptake (Acevedo et al., 2014). Consequently, the complexity of metabolic models, in terms of stoichiometric and kinetic descriptions of the underlying processes, as well as the number of different organisms to account for, has been on the rise. This trend is set to continue with the incorporation of newly-recognized putative PAO and GAO, e.g., *Tetrasphaera* (Maszenan et al., 2000), more detailed characterization of GAO metabolisms, namely glycolysis pathways and VFA-uptake mechanisms, as well as correct differentiation of alternative metabolic pathways from the presence of different bacterial strains (Oehmen et al., 2010a).

This ever-increasing complexity and sprawling-nature of metabolic models must however be justified, given the level of detail and number of organisms already described. Variations in model predictions could be exacerbated by uncertainty associated with the input parameters (Sin et al., 2005). Therefore, this work seeks to address for the first time, in a comprehensive way, the subjective uncertainty in metabolic models for EBPR, i.e., that arising from incomplete knowledge about the true value of the model's input parameters, by performing a global sensitivity analysis (GSA) to determine the key parameters that influence their predictions.

Although previous studies reported local sensitivity analyses, such procedures consider the variation of different input parameters only one at a time and typically at the same initial conditions, e.g., in Lopez-Vazquez et al. (2009), Oehmen et al. (2010b), and Lanham et al. (2014). As such, they do not account for interactions between different sources of uncertainty, apart from the case of simple linear models (Saltelli et al., 2006). In contrast, GSA seeks to ascertain the relative importance of key processes and/or parameters driving the output dynamics of the overall system by perturbating all inputs simultaneously. It has been used effectively in areas of risk assessment (Mokhtari and Frey, 2005), experimental design (Kent et al., 2013) and model development (Sin et al., 2009) applied to biochemical systems.

GSA provides a basis for the justification (or rejection) of previous assumptions regarding the estimation of input parameters during model calibration, thereby generating feedback with which to improve existing model formulations. Most importantly, GSA enables meaningful comparisons of different model formulations by decoupling uncertainty in the required input parameters from the heterogeneity in the calibration procedure. In this study, a mechanistic model was developed to integrate available knowledge concerning the metabolism of *Accumulibacter* PAO in anaerobic-aerobic EBPR systems. Given the model's complexity, underlying assumptions and uncertainty of the true value of its various input parameters, a GSA was conducted by simultaneous perturbation of all input parameters.

First, standard regression coefficients (SRC) (Saltelli et al., 2008) were determined from the ordinary least squares (OLS) method using linear regression on the output of Monte Carlo simulations. Alternative regression methods, e.g., step-wise, ridge or least absolute shrinkage and selection operator (LASSO) were considered, owing to the additional step of selecting predictor variables that aid in the interpretation of the fitted model (Tibshirani, 1996). Nevertheless, regression by OLS was chosen as the most fit-for-purpose, given that (1) sampling of the input parameter space was randomized (low collinearity), (2) the overall number of sample points was large relative to the number of predictor variables, and (3) the consequences of over-fitting were negligible, thereby negating the advantages of more sophisticated methods (Melkumova and Shatskikh, 2017).

SRC results were complemented with sensitivity indices obtained via the Sobol method for variance decomposition to investigate non-linear interactions (Sobol, 2001). Furthermore, Sobol sensitivity indices were also computed to determine the combined influence of multiple-inputs to reveal higher-order interactions (Saltelli et al., 2008). Although alternative variance-based methods are available, e.g., the Fourier amplitude sensitivity test (FAST), which can be computationally more efficient, the Sobol method was selected on account of being more robust, particularly for the determination of total-order sensitivities (Saltelli and Bolado, 1998).

The contribution of each input parameter's uncertainty on the variance of model outputs was then calculated. In this way, input parameters that most significantly affect the model output were identified. Opportunities for factor fixing and model simplification were investigated in view of reducing the complexity of existing models, thereby facilitating parameter estimation their integration within the ASM framework. This would ensure that the limitations and uncertainty intervals of current metabolic models would be better understood. In addition, it can flag key parameters that carry the greatest uncertainty, which would need careful measurement or further experimental investigation. Models would become more applicable to the general case rather than being specific to any particular experimental design, which could significantly improve their homogeneity and applicability. This would ultimately lead to improved efficiency and reliability of EBPR systems, an important pollution control and resource recovery technology.

## 2. Materials and Methods

### 2.1. Model Construction, Evaluation, and Analysis Environment

Data processing and analysis were done in the Python programming language, ver 3.7. This included: (1) characterization of input uncertainty, (2) sampling from the space of input factors, (3) propagation of input uncertainty to the output and (4) quantifying the contribution of input uncertainty on the variance of model outputs. Where possible, parts of the implementation were adapted from SALib ver 1.1.2 (Herman and Usher, 2017). Python script files can be made available upon request.

### 2.2. Model Description

The model described the anaerobic-aerobic metabolism of *Accumulibacter* PAO. Stoichiometric and kinetic dependencies on the carbon source as acetate (HAc) and propionate (HPr) were implemented according to Smolders et al. (1995) and Oehmen et al. (2005b), respectively. Modifications to the maximum substrate uptake rates were implemented in accordance with Carvalheira et al. (2014b). The dependence of intra-cellular poly-hydroxyalkanoate (PHA) formation on carbon source followed that of Zeng et al. (2003b). Temperature dependencies were accounted for as described in Brdjanovic et al. (1997). The effect of pH on stoichiometric yields and kinetics was implemented in line with Filipe et al. (2001a) and Filipe et al. (2001c), respectively. Sequential maintenance on PHA, glycogen and poly-P followed Lanham et al.'s (2014) formulation.

Overall, the model tracked one 5 h operational cycle, split between an anaerobic and aerobic phase of equal duration, supplied with a mixture of HAc and HPr. These included 4 dissolved components in the bulk liquid phase: O_2_, HAc HPr and PO_4_. It also accounted for the concentration of PAO biomass, alongside its intra-cellular storage compounds: poly-β-hydroxybutyrate (PHB), poly-β-hydroxyvalerate (PHV), poly-β-hydroxy-2-methylvalerate (PH_2_MV), glycogen and poly-P. The process of biomass growth was described as the difference between total PHA degradation and that used for the replenishment of poly-P and glycogen reserves (Murnleitner et al., 1997; Lopez-Vazquez et al., 2009). In aggregate, the model consisted of 10 components and 10 kinetic processes (see Appendices 1, 5 in Supplementary Materials).

### 2.3. Characterization of Input Uncertainty

A total of 39 input parameters were considered. These were allocated into four categories for analysis: metabolic parameters, kinetic parameters, Arrhenius temperature coefficients and initial conditions. Metabolic parameters included: the ATP requirement for biomass synthesis from Acetyl-CoA (*K*_1_) and Propionyl-CoA (*K*_2_), the yield of ATP per unit of NADH_2_ oxidized (δ), the phosphate transport coefficient (ε) and the half-saturation constants (*K*_HAc_, *K*_HPr_, *K*_P_O__4__, *K*_PHA_, *K*_Gly_, *K*_f_PHA__, *K*_PP_). Kinetic parameters included the maximum specific rates of anaerobic HAc (*q*_HAc_) and HPr (*q*_HPr_) uptake, the anaerobic maintenance coefficient (*m*_ATP, an_), as well as the rates of aerobic PHA degradation (*q*_PHA_), glycogen production (*q*_Gly_), poly-P formation (*q*_PP_) and the aerobic maintenance coefficient (*m*_ATP, ox_). The maximum intra-cellular fractions of glycogen (*f*_Gly, max_) and poly-P (*f*_PP, max_) were also regarded as kinetic parameters. Arrhenius temperature coefficients included those corresponding to the effect of temperature on: anaerobic uptake of HAc and HPr (θ_*q*_VFA__), anaerobic maintenance (θ_*m*_ATP, an__), as well as aerobic PHA degradation (θ_*q*_PHA__), glycogen production (θ_*q*_Gly__), poly-P formation (θ_*q*_PP__) and aerobic maintenance (θ_*m*_ATP, ox__). Finally, initial conditions, i.e., the total concentration of carbon sources *S*_VFA, *i*_ and the corresponding fraction of HAc (*r*_HAc/HPr, *i*_) in the influent, the ratio of PO_4_ to carbon (*r*_P/C, *i*_), the initial biomass concentration (*X*_PAO, *i*_), as well as the intra-cellular fractions of PHA (*X*_PHB, *i*_, *X*_PHV, *i*_, *X*_P_H__2_MV, *i*_), glycogen (*X*_Gly, *i*_) and poly-P (*X*_PP, *i*_) were also taken into consideration. As the distribution from which the input parameters were sampled was not uniform, readers are directed to the Supplementary Materials for a detailed account.

Input uncertainty was characterized by systematic review of quantities whose values had been measured or otherwise estimated from experimental data in the literature. Each parameter was considered a random variable that followed a uniform distribution between a minimum and maximum value of *a* and *b*, respectively. These ranges were selected based on the degree of variation, *var*, around the average, *x*_*mean*_. Variability was scaled to reflect the number of data reported in the literature, as 50, 25, or 5% for 1, less or equal to 10, or more measurements, respectively. To ensure that the range extended at least to the limits found in the literature, the minimum value in the collected data was taken as the lower bound *a* if it was smaller than that determined using *var*. The upper bound was selected in a similar manner, as defined by Equations (1) and (2).

(1) $$a=min((1-var)x_{\text{mean}},\;x_{\text{min}})$$

(2) $$b=max((1+var)x_{\text{mean}},\;x_{\text{max}})$$

To conform to bounds within which the model has been experimentally validated, temperature was sampled between the limits of 10 to 30°C, and pH was sampled between 6 and 7.5. To reflect the nature of knowledge completeness regarding the value of parameters where sufficient experimental data could be obtained (i.e., > 50 points), the data was fitted to the Erlang distribution.

Model inputs were generated via Monte Carlo sampling, where each *X*_*i*_ constituted a set of input parameter values with which to evaluate the model:

(3) $$X_{i}=[x_{1,i},x_{2,i},\ldots x_{K,i}]\text{ for }i=1,2,\ldots N$$

where *N* is the total number of samples. Initial concentrations in the bulk liquid phase, biomass and intra-cellular components were also included as part of the input uncertainty, given that characterization of the influent to a sufficient level of detail is a known obstacle. In this sense, the initial concentrations of HAc, HPr and PO_4_ were derived as ratios from the total concentration of carbon substrate. Intra-cellular fractions of PHB, PHV and PH_2_MV were sampled according to the Dirichlet distribution, in order to ensure that their sum would be equal to the total PHA fraction, which itself followed the uniform distribution. The implementation was based on Saltelli et al.'s (2008, 2010) extension of Sobol sequences to generate quasi-random points.

The *N* × *M* dimensional matrix of inputs, where *M* is the total number of parameters was propagated via the metabolic model, yielding a three-dimensional matrix of outputs *Y*_*T* × *K* × *N*_, where *T* is the number of time steps and *K* is the number of output variables. The mean of the concentration profile over one cycle was calculated, as the methods for quantifying the sensitivity indices require scalar values.

### 2.4. Sensitivity Analysis Measures

#### 2.4.1. Standard Regression Coefficients

Standard regression coefficients were obtained by performing a series of linear regressions on data from the matrix of inputs and each column of the output matrix:

(4) $$y_{i,k}=b_{m}+\sum_{m=1}^{M}b_{m,k}x_{i,m}+\epsilon_{i,k}\text{ for }i=1,2,\ldots N\text{ and }k=1,2,\ldots K$$

where *y*_*i, k*_ is the vector of values corresponding to the *kth* output variable, *b*_*m, k*_ is the coefficient of the *mth* input parameter, *x*_*i, m*_ is the value of the *m*^*th*^ parameter and ϵ_*i, k*_ is the residual error. As shown in Equation (5), the standard regression coefficient β_*m, k*_, i.e., the effect of parameter *m* on output *k*, relative to all other parameters, was determined by scaling the input and output values by their respective means and standard deviations:

(5) $$\frac{y_{i,k}-\mu_{k}}{\sigma_{k}}=\sum_{m=1}^{M}\beta_{m,k}\frac{x_{i,k}-\mu_{m}}{\sigma_{m}}+\epsilon_{i,k}$$

#### 2.4.2. Sobol Method for Variance Decomposition

The variance observed in the output can be expressed as the sum of variances of individual parameters and their combinations, as shown in Equations 6 to 8 (Saltelli et al., 2008):

(6) $$Var(Y)=\sum_{i=1}^{M}V_{i}+\sum_{i<j}^{M}V_{ij}+\ldots +V_{1,2,\ldots ,m}$$

(7) $$V_{i}=V_{X_{i}}(E_{X_{i}}(Y|X_{i}))$$

(8) $$V_{ij}=V_{X_{ij}}(E_{X_{\sim ij}}(Y|X_{i},X_{j}))-V_{i}-V_{j}$$

where *Var*(*Y*) is the overall variance of the output, *V*_*i*_ is the variance of the input parameter *i* and *V*_*ij*_ is the partial variance of parameters *i* and *j*. *X*_*i*_ denotes the set values taken by parameter *i*, and *X*_~*i*_ denotes the set of values taken by all parameters except *i*.

(9) $$S_{i}=\frac{V_{i}}{Var(Y)}$$

(10) $$S_{Ti}=\frac{E_{X_{\sim i}}\left(Var\left(Y|X_{\sim i}\right)\right)}{Var(Y)}=1-\frac{Var_{X_{\sim i}}\left(E_{X_{i}}\left(Y|X_{\sim i}\right)\right)}{Var(Y)}$$

From this, the first-order sensitivity index, also referred to as the “effect” of a given parameter *i*, can be calculated as shown in Equation (9). The total effect of parameter *i*, including the direct and interactions with other parameters can be expressed according to Equation (10). Numerical estimation of the first and total-order Sobol indices was implemented according to Saltelli et al. (2010).

## 3. Results

### 3.1. Quantification of Output Uncertainty Using Monte Carlo Simulations

**Figure 1:**
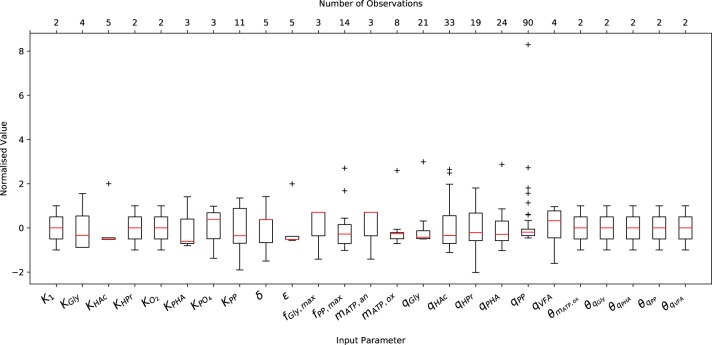
Variation in the measured or predicted values of the input parameters normalized by their respective mean and standard deviation. The boxplot includes the normalized median (red horizontal line within the box), interquartile range (IQR), i.e., from 25th to 75th percentile (box), 1.5 times the lowest and highest percentiles (whiskers) and the number of observations (upper x-axis).

A comprehensive literature review was conducted on relevant articles from 1993 to 2017 to characterize the variance of experimentally measured (or indirectly estimated from experimental data) values of the input parameters necessary to initialize the metabolic model. As shown in Figure 1, both the abundance and the minimum and maximum range of the data varied considerably from one parameter to another. Note that Figure 1 only shows the variation of input parameters that have been reported in the literature, and does not include (single) point values, e.g., the ATP yield per unit of biomass on Propionyl-CoA (*K*_2_) and the half-saturation constant for propionate uptake (*K*_HPr_), nor the characteristic variation of initial conditions. The underlying data was normalized by subtracting the mean and dividing by the corresponding standard deviation to illustrate the variance, rather than the difference in magnitude. In general, more complete data was obtained for kinetic parameters, namely the maximum specific rates of anaerobic HAc uptake (*q*_HAc_) and aerobic Poly-P formation (*q*_PP_), for which 33 and 90 measurements were reported, respectively.

Only 2 measurements were available for the Arrhenius temperature coefficients, as only two studies have quantified the short and long-term temperature dependencies (Brdjanovic et al., 1997, 1998). The sparsity of data for the half-saturation coefficients results from the fact that the same values have been reused in nearly all previous studies concerning EBPR metabolic models. This is likely due to the assumption that, as in ASM models, they do not deviate to any appreciable extent. In the case of metabolic parameters, namely the yield of ATP per unit of NADH_2_ (δ) and the phosphate transport coefficient (σ), the small number of measurements likely arises from the difficulty in measuring these quantities experimentally. While another factor may be that their deviation from the mean has had no appreciable effect on model predictions, as demonstrated in Zeng et al. (2003b), this conclusion was drawn from a local sensitivity analysis. Whether this conclusion holds for initial conditions other than those applied in the aforementioned study has yet to be determined.

**Figure 2:**
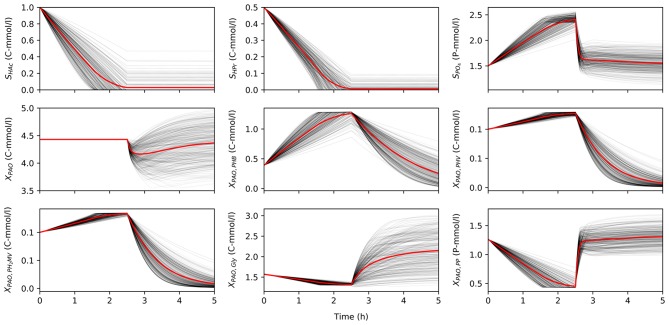
The variance of model predictions given the uncertainty of the input parameters for one set of initial conditions in a 5 h anaerobic-aerobic cycle (2.5 h each). Mean of the concentration profile is highlighted in red.

Once characterized, the input uncertainty was propagated through the model to obtain a set of concentration profiles for each permutation of input parameters and initial conditions. Figure 2 shows a subset obtained by varying the input parameters, while holding the initial conditions constant to illustrate the variance in model predictions due to input uncertainty. Specifically, the total concentration of VFA (*S*_VFA, *i*_) in the influent was 1.5 C-mmol/l, the ratio of HAc to HPr (*r*_HAc/HPr, *i*_) was 0.67, and the ratio of PO_4_ to VFA (*r*_P/C, *i*_) was 1.5. The initial concentration of biomass was 4.43 C-mmol/l, and the initial fractions of PHA (*X*_PHA, *i*_), glycogen (*X*_Gly, *i*_), and poly-P (*X*_PP, *i*_) were 0.14, 0.36, and 0.3, respectively, in the first part of this study. When HAc and HPr were not fully consumed in the anaerobic phase, the model disregarded aerobic consumption of carbon substrates, so as not to influence the model outputs with other aerobic processes due to aerobic consumption of VFA.

The uncertainty of model predictions varied from one output variable to another. In addition, the output variance was also dependent on time, in line with changing redox conditions. Most notably, the concentration of biomass was insensitive to any input uncertainty in the anaerobic phase, since according to the model no growth is expected in these conditions, but diverges significantly in the aerobic phase. A similar trend was observed for concentration profiles of intra-cellular glycogen (*X*_Gly_).

The intra-cellular concentrations of PHV and PH_2_MV were less sensitive in the anaerobic compared to the aerobic phase, whereas the concentration of PHB showed greater variance in both redox environments. However, it must be noted that PHB was also the most abundant fraction of intra-cellular PHA, and one that results from the metabolism of both HAc and HPr, whereas PHV results mainly from the metabolism with HPr. Consequently, the low variance in the final value of PHV and PH_2_MV at the end of the anaerobic phase was likely due to these factors aggravated by the lower *S*_HPr, *i*_. Likewise, the lower variance at the end of the aerobic phase results from the near complete depletion of all PHA components. As shown in Figure 2, the final concentration of PHV and PH_2_MV in the majority of simulations was below 0.1 C-mmol/l.

**Figure 3:**
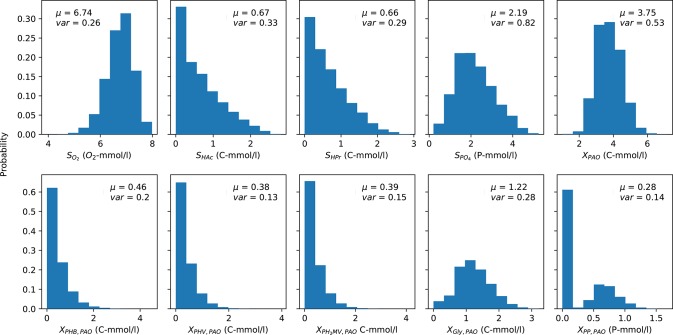
Distribution of the average PAO model predictions over one cycle for each output variable obtained via Monte Carlo simulations. μ and *var* indicate the mean and variance of the output, respectively. The y-axis indicates the fraction of occurrences corresponding to a particular outcome from 0 to 1.

The mean (over time) of the PAO output concentration profiles for each complete anaerobic-aerobic cycle are summarized in the form of histograms in Figure 3. These were obtained by taking into account the uncertainty associated with the input parameters alongside the initial conditions, e.g., concentration of carbon sources in the influent, the prevailing temperature, pH and the starting content of intra-cellular polymers in the biomass cells, for a total of 164,000 input samples. While there is considerable variance for all model outputs, the distribution of the concentrations of carbon compounds (extra-cellular VFA and intra-cellular PHA) were skewed toward the lower end while the rest were centered around a particular mean. The mean concentration profiles of phosphate in the bulk liquid phase and biomass were subject to the highest degree of variance, whereas the concentrations of intra-cellular PHV, poly-P and PH_2_MV deviated the least under different permutations of input parameters. The mean intra-cellular poly-P concentration (*X*_PP_) was the only variable with a bi-modal distribution, with a value near zero in over half of the outcomes. Based on the skewness of the final outcomes, it can be expected that the coefficient of determination, as a proxy of the validity of SRC, would also suffer.

### 3.2. Sensitivity Analysis

#### 3.2.1. First-Order Effects

Two methods were used to assess the first-order effects of each input parameter on the output parameters: estimation of SRC and the Sobol method for variance decomposition. Unlike local sensitivity analyses, these methods assess the effect of a given input parameter on an output variable under all possible permutations of different inputs.

For a linear model, the square of the SRC, i.e., β^2^, would be equal to the corresponding first-order index obtained via the Sobol method (see section 2.3.2). The value of β lies between -1 and +1, indicating both the direction as well as the magnitude of the effect of a given input parameter on the output variable. However, the robustness of this sensitivity measure is dependent on the degree of linearity between the input with respect to the output. As such, the validity of SRC as an estimate of the first-order effect depends on the coefficient of determination (*R*^2^). As a rule of thumb, this value should be ≥ 0.7 for the variance of the output variable to be sufficiently correlated to the variance of the input parameter (Campolongo and Saltelli, 1997). Although the sum of all β^2^ with respect to a given output for a linear model would be equal to 1, owing to the non-linear form of the underlying equations in this work, it was expected to be less than one (Saltelli et al., 2008).

**Figure 4:**
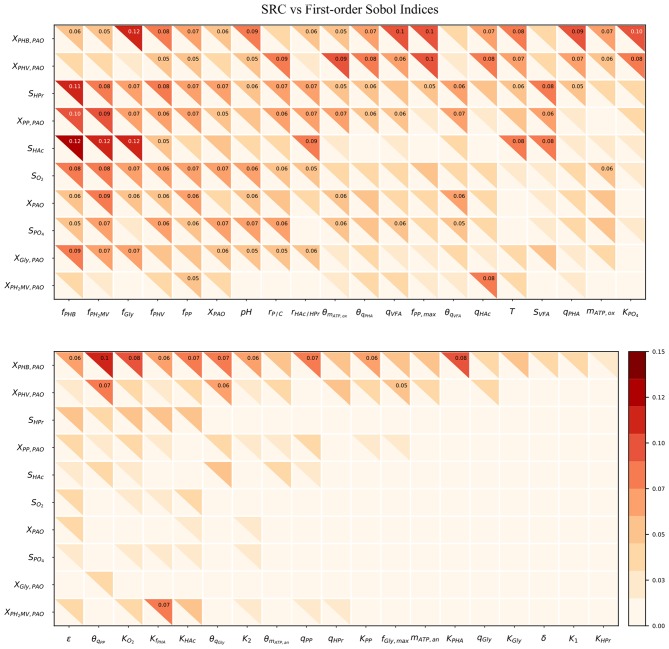
Heatmap of the SRC and first-order Sobol indices for PAO-related input parameters. Rows indicate output variables, whereas columns indicate input parameters. SRC values are located in the lower-left triangle. The first-order Sobol index is in the upper-right triangle. The cut-off value to display the sensitivity index was 0.05.

Figure 4 compares the β^2^ coefficients and first-order Sobol sensitivity indices as estimates of the direct effect of each input on a given output. The higher the value of the index, the higher the effect of that input parameter on the corresponding output parameter relative to the other inputs. Specifically, the Sobol index indicates the relative degree of output that arises from the uncertainty the input in question. The input parameters were listed in order of greatest to lowest influence on the model output uncertainty, reading from left to right. Likewise, the output variables were ordered from most to least uncertain, reading from top to bottom.

The first-order Sobol index was found to be greater than that of the SRC in every instance. This was in line with expectations, as the calculation of the first-order Sobol index does not rely on the assumption of linearity, and is therefore able to capture non-linear effects as well. Furthermore, the *R*^2^ corresponding to each output ranged from only 0.006 to 0.018, indicating that SRC is not an appropriate sensitivity measure for this metabolic model. This suggests that model simplification by linearization of any part of the system of differential equations by which model predictions are defined is not a feasible option as long as accurate predictions are desired in the operating range specified by the input uncertainty.

As shown by the first-order Sobol indices, the most influential parameters on the prediction of concentrations in the bulk liquid phase were the initial concentration of PAO biomass (*X*_PAO, *i*_) and the intra-cellular concentrations of PHA constituents (defined in this study as fractions of the total initial PHA concentration, *f*_PHB, *i*_, *f*_PHV, *i*_ and *f*_P_H__2_MV, *i*_), followed by that of intra-cellular glycogen (*f*_Gly, *i*_). That the variance of components in the liquid phase depends on the concentration of PAO is a given, as the rate at which substrates are utilized is directly proportional to the number of organisms. The importance of intra-cellular PHA and glycogen, particularly on the prediction of dissolved oxygen (*S*_O__2_) and VFA concentrations is explained by their role in the transport and storage of carbon sources to survive in alternating feast-famine conditions, serving as sources of energy and reducing agents for substrate uptake, growth and cell maintenance. Table 1 presents a more specific ranking of the influential input parameters for each output.

**Table 1:** Input parameters ranked according to the most to the least influential with respect to each of the model output variables, as determined by the first-order Sobol sensitivity index.

***S*_O__2_**	***S*_HAc_**	***S*_HPr_**	***S*_PO__4_**	***X*_PAO_**	***X*_PHB_**	***X*_PHV_**	***X*_PH__2MV_**	***X*_Gly_**	***X*_PP_**
*f*_PH_2_MV, *i*_	*f*_PHB, *i*_	*f*_PHB, *i*_	pH	*f*_PH_2_MV, *i*_	*f*_Gly, *i*_	*f*_PP, max_	*q*_HAc_	*f*_PHB, *i*_	*f*_PHB, *i*_
*f*_PHB, *i*_	*f*_Gly, *i*_	*S*_VFA, *i*_	*X*_PAO, *i*_	*f*_PP, *i*_	*K*_P_O__4__	θ_*m*_ATP, ox__	*K*_*f*_PHA__	*f*_PH_2_MV, *i*_	*f*_PH_2_MV, *i*_
*f*_PP, *i*_	*f*_PH_2_MV, *i*_	*f*_PHV, *i*_	*f*_PH_2_MV, *i*_	θ_*q*_VFA__	θ_*q*_PP__	*r*_P/C, *i*_	*f*_PP, *i*_	*f*_Gly, *i*_	θ_*m*_ATP, ox__
*X*_PAO, *i*_	*r*_HAc/HPr, *i*_	*f*_PH_2_MV, *i*_	*f*_PHV, *i*_	*f*_PHV, *i*_	θ_*q*_VFA__	θ_*q*_PHA__		*r*_HAc/HPr, *i*_	*f*_Gly, *i*_
*f*_Gly, *i*_	T	*X*_PAO, *i*_	*r*_P/C, *i*_	*f*_Gly, *i*_	*f*_PP, max_	*q*_HAc_		*X*_PAO, *i*_	θ_*m*_ATP, ox__
*f*_PHV, *i*_	*S*_VFA, *i*_	*r*_P/C, *i*_	*q*_VFA_	pH	*q*_PHA_	*K*_P_O__4__		pH	*r*_HAc/HPr, *i*_
pH	*f*_PHV, *i*_	*f*_Gly, *i*_	θ_*m*_ATP, ox__	θ_*m*_ATP, ox__	pH	*q*_PHA_		*r*_P/C, *i*_	*f*_PP, *i*_
*r*_P/C, *i*_	*X*_PAO, *i*_	*r*_HAc/HPr, *i*_	*f*_PP, *i*_	*f*_PHB, *i*_	*K*_PHA_	T			*r*_P/C, *i*_
*m*_ATP, ox_		*f*_PP, *i*_	*f*_PHB, *i*_		*K*_O2_	θ_*q*_PP__			*f*_PHV, *i*_
*r*_HAc/HPr, *i*_		θ_*q*_VFA__	θ_*q*_VFA__		*f*_PHV, *i*_	*q*_VFA_			*S*_VFA, *i*_
		θ_*q*_PHA__			T	θ_*q*_Gly__			*q*_VFA_
		T			*K*_HAc_	*m*_ATP, ox_			θ_*q*_VFA__
		pH			*f*_PP, *i*_	*f*_PHV, *i*_			*X*_PAO, *i*_
		θ_*m*_ATP, ox__			θ_*q*_Gly__	*f*_PP, *i*_			
		*q*_PHA_			*q*_HAc_	*f*_Gly, max_			
		*f*_PP, max_			*m*_ATP, ox_	pH			
		*q*_HAc_			*q*_PP_				
					θ_*q*_PHA__				
					*K*_*f*_PHA__				
					*K*_PP_				
					ε				
					*X*_PAO, *i*_				
					*r*_HAc/HPr, *i*_				
					*K*_2_				
					*f*_PHB, *i*_				
					*f*_PH_2_MV, *i*_				
					θ_*m*_ATP, ox__				

*Input parameters whose sensitivity was lower than the average are not shown*.

Intra-cellular glycogen ranked highly for all liquid phase outputs apart from the PO_4_ concentration. The lower influence of *f*_Gly, *i*_ on the prediction of *S*_P_O__4__ compared to that of intra-cellular PHA can be explained by the degree of relatedness of the processes in question. While PO_4_ uptake for poly-P formation under aerobic conditions is directly dependent on intra-cellular PHA reserves, it is only indirectly dependent on the intra-cellular glycogen concentration, where the availability of PHA is affected by glycogen content in the biomass for PHA formation in the preceding anaerobic phase.

The pH was highly influential with respect to the prediction of all liquid phase concentrations, most notably that of *S*_PO__4_. The one exception was *S*_HAc_. While seemingly surprising, as pH is linked directly to the amount of energy ( ATP) required to transport VFA across the cell membrane, this discrepancy could be explained by its involvement in the stoichiometric yield of PHV and PH_2_MV formation (as opposed to the more straightforward conversion to PHB), in addition to glycogen and poly-P degradation during anaerobic PO_4_ release in the presence of HPr. In contrast, the stoichiometry with respect to anaerobic glycogen degradation is independent of pH when HAc is the primary VFA.

Although temperature ranked among the most influential input parameters with respect to the intra-cellular concentration of PHB (*X*_PHB_), *S*_HAc_ and *S*_HPr_, it did not do so consistently for the remaining outputs. As shown in Table 1, it is uncertainty regarding the Arrhenius coefficients (θ_*i*_) that drives the variance in model outputs in general. When both temperature and Arrhenius coefficient are influential, with the exception of *S*_HAc_, the Arrhenius coefficient is more important. Although seemingly surprising, given comparable variances of the corresponding input uncertainties (50% around the mean), this discrepancy may be due to the exponential form of the temperature dependence of the kinetic processes. In general, the Arrhenius coefficient for VFA uptake (θ_*q*_VFA__), PHA formation (θ_*q*_PHA__) and poly-P formation (θ_*q*_PP__) are more influential for all outputs. The Arrhenius coefficient corresponding to aerobic maintenance (θ_*m*_ATP, ox__) ranks notably high with respect to the prediction of *X*_PAO_, the intra-cellular concentration of PHV (*X*_PHV_), *S*_P_O__4__ and the concentration of intra-cellular poly-P (*X*_PP_).

#### 3.2.2. Comparing First-Order and Total-Order Effects

The Sobol method decomposes the variance of the output variables (or sets of variables) into fractions attributable to either individual input parameters or sets of inputs. Unlike SRC, the resultant sensitivity indices take into account non-linear responses, as well as interactions between different input parameters (Saltelli and Annoni, 2010). The total-order sensitivity index quantifies the aggregate influence of a given input parameter, inclusive of its interaction with all other inputs on a given output variable.

**Figure 5:**
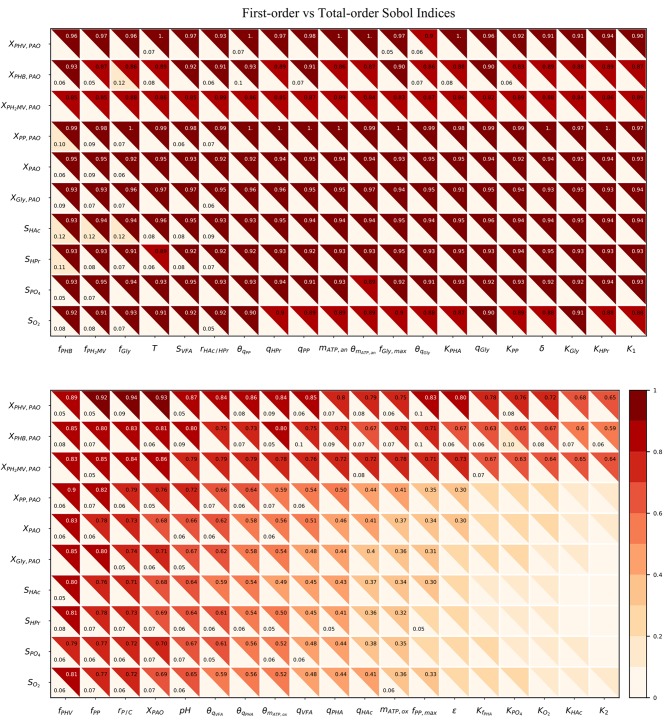
Heatmap of the first and total-order Sobol indices. Rows represent output variables and columns represent input parameters. The first-order effect is in the lower-left triangle. The total-order index is in the upper-right triangle. The cut-off values to display the first and total-order sensitivities were 0.05 and 0.3, respectively.

As shown in Figure 5, the total-order sensitivity was found to be greater than that of the first-order in all instances. This, coupled with the fact that the confidence interval was on average approximately 8% of the sensitivity index, confirms that the total-order indices were an accurate apportionment of the input uncertainties to the output variance (Saltelli et al., 2010). Although the relative influence of the input parameters on the output variables was identical for both first and total-order sensitivities the magnitude of the effect was more pronounced in the latter. Whereas the first-order indices ranged from 0 to 0.12, the maximum value of the total-order indices was 1. Differences between the first and sensitivities show that the major cause of variance in the model outputs was not the uncertainty of individual parameters. Rather, it was the interaction among the inputs, revealed by simultaneous perturbation of input parameters using the Monte Carlo method.

As seen from the lower-right quadrant of Figure 5, the total effect of the half-saturation constants for *f*_PHA_, PO_4_, O_2_, HAc, as well as the yield of ATP per unit of biomass on propionyl-CoA (*K*_2_) and the aerobic phosphate transport coefficient (ε) on the output variables were relatively small. The exception to this trend were the intra-cellular PHA concentrations : *X*_PHB_, *X*_PHV_ and *X*_P_H__2_MV_. However, as the distribution of the total effect was relatively uniform beyond the limits of this quadrant, it was difficult to assess opportunities for model simplification based solely on the raw values of the Sobol indices. In contrast to the first-order sensitivities, it would no longer be feasible to assign fixed values to the aforementioned input parameters, as their influence on the concentration of intra-cellular PHA components are not negligible when higher order interactions are taken into account.

#### 3.2.3. Relative Contribution of Total Effects

**Figure 6:**
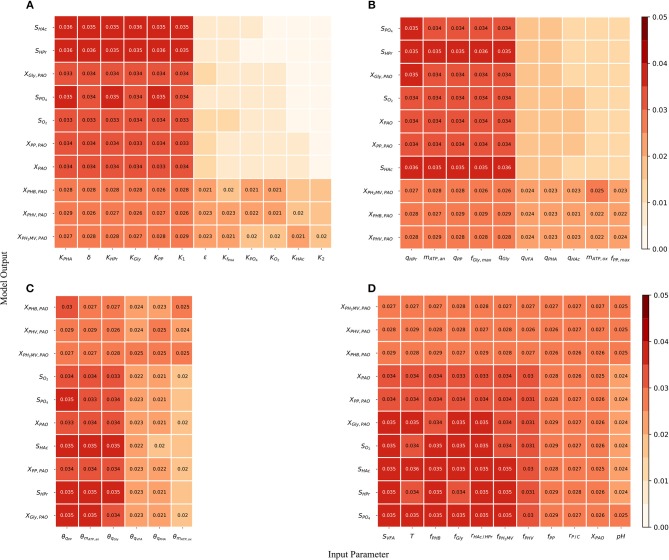
**(A)** Normalized total effect of the half-saturation coefficients and metabolic yields on the model outputs. **(B)** Normalized total effect of the kinetic parameters on the model outputs. **(C)** Normalized total effect of the Arrhenius temperature coefficients on the model outputs. **(D)** Normalized total effect of the initial conditions on the model outputs.

Although the total-order indices were large in many cases, their significance must be taken in the context of the other parameters. It is known that for a linear model the sum of the first-order Sobol indices must be equal to 1. Further, the first-order sensitivities would be equal to the total-order ones in the absence of interactions. Based on this notion, the total-order indices for each output variable were normalized such that their sum would be equal to 1. The data was then separated according to four principal groups of input parameters: (1) metabolic parameters, (2) kinetic parameters, (3) Arrhenius temperature coefficients and (4) initial process conditions. The corresponding heat-maps of the transformed total-order sensitivity indices for each group are shown in Figures 6A–D. Table 2, discussed in section 4, presents the most influential inputs with respect to each output variable.

**Table 2:** Input parameters ranked according to the most to the least influential with respect to each of the model output variables, as determined by the total-order Sobol sensitivity index.

**Group**	***S*_O__2_**	***S*_HAc_**	***S*_HPr_**	***S*_PO__4_**	***X*_PAO_**	***X*_PHB_**	***X*_PHV_**	***X*_PH__2MV_**	***X*_Gly_**	***X*_PP_**
Metabolic	*K*_Gly_	*K*_Gly_	δ	*K*_HPr_	*K*_Gly_	δ	*K*_PHA_	*K*_1_	*K*_Gly_	δ
	*K*_PP_	*K*_PHA_	*K*_PHA_	*K*_PP_	*K*_PHA_	*K*_HPr_	*K*_HPr_	*K*_PP_	*K*_PP_	*K*_HPr_
	*K*_HPr_	δ	*K*_PP_	*K*_PHA_	δ	*K*_Gly_	*K*_PP_	δ	*K*_1_	*K*_PP_
	*K*_1_	*K*_1_	*K*_HPr_	*K*_1_	*K*_HPr_	*K*_PHA_	*K*_Gly_	*K*_HPr_	δ	*K*_PHA_
	δ	*K*_PP_	*K*_Gly_	δ	*K*_1_	*K*_1_	δ	*K*_PHA_	*K*_HPr_	*K*_Gly_
	*K*_PHA_	*K*_HPr_	*K*_1_	*K*_Gly_	*K*_PP_	*K*_PP_	*K*_1_	*K*_Gly_	*K*_PHA_	*K*_1_
Kinetic	*q*_Gly_	*q*_Gly_	*f*_Gly, max_	*q*_HPr_	*q*_PP_	*q*_PP_	*m*_ATP, an_	*m*_ATP, an_	*q*_HPr_	*q*_PP_
	*f*_Gly, max_	*q*_HPr_	*m*_ATP, an_	*m*_ATP, an_	*q*_HPr_	*f*_Gly, max_	*q*_PP_	*q*_PP_	*q*_PP_	*q*_HPr_
	*q*_HPr_	*f*_Gly, max_	*q*_Gly_	*q*_Gly_	*m*_ATP, an_	*q*_Gly_	*q*_HPr_	*q*_HPr_	*m*_ATP, an_	*m*_ATP, an_
	*m*_ATP, an_	*m*_ATP, an_	*q*_HPr_	*f*_Gly, max_	*q*_Gly_	*q*_HPr_	*f*_Gly, max_	*f*_Gly, max_	*q*_Gly_	*f*_Gly, max_
	*q*_PP_	*q*_PP_	*q*_PP_	*q*_PP_	*f*_Gly, max_	*m*_ATP, an_	*q*_Gly_	*q*_Gly_	*f*_Gly, max_	*q*_Gly_
Arrhenius coef	θ_*q*_PP__	θ_*m*_ATP, an__	θ_*m*_ATP, an__	θ_*q*_PP__	θ_*q*_Gly__	θ_*q*_PP__	θ_*m*_ATP, an__	θ_*q*_Gly__	θ_*m*_ATP, an__	θ_*q*_PP__
	θ_*m*_ATP, an__	θ_*q*_Gly__	θ_*q*_Gly__	θ_*q*_Gly__	θ_*m*_ATP, an__	θ_*m*_ATP, an__	θ_*q*_PP__	θ_*q*_PP__	θ_*q*_PP__	θ_*m*_ATP, an__
	θ_*q*_Gly__	θ_*q*_PP__	θ_*q*_PP__	θ_*m*_ATP, an__	θ_*q*_PP__	θ_*q*_Gly__	θ_*q*_Gly__	θ_*m*_ATP, an__	θ_*q*_Gly__	θ_*q*_Gly__
Process condition	*f*_Gly, *i*_	T	*f*_PHB, *i*_	*f*_PH_2_MV, *i*_	*f*_PH_2_MV, *i*_	*f*_PHB, *i*_	T	*r*_HAc/HPr, *i*_	*S*_VFA, *i*_	*f*_Gly, *i*_
	*r*_HAc/HPr, *i*_	*S*_VFA, *i*_	*f*_PH_2_MV, *i*_	*S*_VFA, *i*_	T	*S*_VFA, *i*_	*f*_PH_2_MV, *i*_	*f*_Gly, *i*_	T	*f*_PHB, *i*_
	*S*_VFA, *i*_	*f*_Gly, *i*_	*S*_VFA, *i*_	*f*_Gly, *i*_	*f*_PHB, *i*_	*r*_HAc/HPr, *i*_	*S*_VFA, *i*_	*X*_PAO, *i*_	*f*_Gly, *i*_	T
	*f*_PHB, *i*_	*f*_PH_2_MV, *i*_	*r*_HAc/HPr, *i*_	*f*_PHB, *i*_	*S*_VFA, *i*_	T	*f*_PHB, *i*_	T	*r*_HAc/HPr, *i*_	*r*_HAc/HPr, *i*_
	*f*_PH_2_MV, *i*_	*f*_PHB, *i*_	*f*_Gly, *i*_	*r*_HAc/HPr, *i*_	*r*_HAc/HPr, *i*_	*f*_PH_2_MV, *i*_	*f*_Gly, *i*_	*f*_PP, *i*_	*f*_PHB, *i*_	*S*_VFA, *i*_
	T	*r*_HAc/HPr, *i*_	T	T	*f*_Gly, *i*_	*f*_Gly, *i*_	*r*_P/C, *i*_	*f*_PHB, *i*_	*f*_PH_2_MV, *i*_	*f*_PH_2_MV, *i*_
	*f*_PHV, *i*_	*f*_PHV, *i*_	*f*_PHV, *i*_	*f*_PHV, *i*_	*f*_PHV, *i*_	*f*_PHV, *i*_	*X*_PAO, *i*_	*S*_VFA, *i*_	*f*_PHV, *i*_	*f*_PHV, *i*_
	*f*_PP, *i*_	*f*_PP, *i*_	*f*_PP, *i*_	*f*_PP, *i*_	*f*_PP, *i*_	*r*_P/C, *i*_	*r*_HAc/HPr, *i*_	*f*_PH_2_MV, *i*_	*f*_PP, *i*_	*f*_PP, *i*_
	*r*_P/C, *i*_	*r*_P/C, *i*_	*r*_P/C, *i*_	*r*_P/C, *i*_	*r*_P/C, *i*_	*X*_PAO, *i*_	*f*_PP, *i*_	*r*_P/C, *i*_	*r*_P/C, *i*_	*r*_P/C, *i*_
	*X*_PAO, *i*_		*X*_PAO, *i*_	*X*_PAO, *i*_			*f*_PHV, *i*_	*f*_PHV, *i*_		*X*_PAO, *i*_
	pH									

*Input parameters whose sensitivity was lower than the average are not shown*.

From Figure 6A, it can be seen that variance in the prediction of liquid phase concentrations, i.e., *S*_O_2__, *S*_HAc_, *S*_HPr_, *S*_P_O__4__, and *X*_PAO_ was due primarily to the uncertainty of a subset of metabolic parameters. This is in contrast to variance in the predition of intra-cellular PHA concentrations, which were due to the uncertainty associated with all metabolic parameters more evenly. Similarly, the prediction of liquid phase concentrations was more sensitive to a smaller subset of kinetic parameters than the prediction of intra-cellular components, specifically the PHA constituents (Figure 6B). The largest difference between the influential and non-influential parameters was found with respect to *S*_HAc_ and *S*_HPr_. This was in line with expectations, as the kinetics of anaerobic VFA uptake tend to be the fastest in the metabolic model of interest. It was curious to note that the effect of the maximum intra-cellular fraction of glycogen (*f*_Gly, max_) was more influential than that of poly-P (*f*_PP, max_) with respect to all outputs. This may be explained by the difference in input uncertainty, as the reported values of *f*_Gly_ deviate more from each other among the different studies.

As shown in Figure 6C and by the ranking in Table 2, the most influential temperature coefficients were those corresponding to anaerobic maintenance (θ_*m*_ATP, an__), aerobic poly-P formation (θ_*q*_PP__) and glycogen formation (θ_*q*_Gly__), respectively. The greatest difference between the influential and non-influential parameters was with respect to the VFA concentration in the liquid phase and the intra-cellular concentration of glycogen (*X*_Gly_).

With regards to the input parameters defining the initial state and the prevailing process conditions, the most influential were the initial intra-cellular fraction of glycogen and PHA, followed by the total VFA concentration in the influent (*S*_VFA, *i*_) and the closely related ratio of HAc to HPr (*r*_HAc/HPr, *i*_), as shown in Figure 6D. Compared with metabolic and kinetic parameters, the contribution of input uncertainties on output variance was spread out more evenly among the Arrhenius temperature coefficients and prevailing process conditions. This indicates that reducing input uncertainty in the model's dependence on temperature and detailed characterization of the influent, inclusive of the intra-cellular components in the biomass, will lead to significant improvements to the accuracy of model predictions with respect to all output variables. In contrast, more careful consideration in the course of parameter prioritization is required for the metabolic and kinetic parameters, given the relatively larger discrepancy between influential and non-influential ones.

## 4. Discussion

### 4.1. Opportunities for Model Simplification Based on First-Order Sensitivities

Linearization of at least parts of the model would result in significantly lower computational cost, and would thereby lower the barrier for the efective use of metabolic models in process monitoring and control. It would also result in more efficient use of resources for the study of more detailed scenarios. This would translate to shorter simulation times and knock-on benefits of the accompanying experimental design, where simulations are used to screen for subsequent laboratory or field work. The validity of SRC depends on the associated *R*^2^ values, which indicate the degree to which interactions between input parameters and output variables can be linearized. By extension, given that metabolic models, particularly those that consider the interaction between multiple species of PAO and GAO are over-parametrized (Yagci et al., 2004), coefficients of determination also indicate the feasibility of model simplification.

Given the small SRC values (< 0.1) and the accompanying *R*^2^ (< 0.018) for all output variables, there is little room for model simplification by linearization of its parts. However, this conclusion is drawn from results obtained by simultaneous perturbation of the model's input parameters, inclusive of the initial conditions. Consequently, although linearization is not feasible for the general case, it may be possible where the initial conditions, i.e., information concerning the concentrations in the bulk liquid phase and the intra-cellular state of PAO cells at the beginning of the anaerobic phase, as well as T and pH, are well-defined and not subject to an appreciable extent of fluctuation from one cycle to another. This can be expected to be the case for laboratory-scale experiments. It may also hold true for wastewater treatment plants where the influent is well characterized and not subject to a significant degree of fluctuation, or if the variation itself is well defined. Based on Figure 2, linearization may be most readily applied to the prediction of HAc and HPr concentrations. It may also be feasible if the system were to be modeled as discrete stages (with redox conditions defining model boundaries), as the predicted behavior follows distinct patterns in the anaerobic and aerobic stages. However, the applicability of such a model would be limited to systems where the redox conditions can be strictly ensured, e.g., no excess of dissolved oxygen in the anaerobic phase.

#### 4.1.1. Influence of Initial Conditions

As shown in Figure 5 and highlighted in Table 1, the most influential parameters tended to describe process conditions, starting with the initial intra-cellular fractions (PHA, glycogen and poly-P), followed by the biomass concentration (*X*_PAO, *i*_), pH, T, and the influent (*r*_P/C, *i*_, *r*_HAc/HPr, *i*_, *S*_VFA, *i*_). It is important to note that the effect of these parameters was typically higher than other metabolic or kinetic parameters. Uncertainty in initial conditions affects the variance of model outputs to a greater degree than input parameters associated with process kinetics or stoichiometry. This finding poses two challenges.

On one hand, if metabolic models are to be applied in full-scale wastewater treatment works, detailed characterization of the influent, namely of the intra-cellular components in biomass, will significantly reduce the variance of all model outputs, leading to more accurate predictions. While characterization of liquid phase components, i.e., the concentrations of HAc, HPr, and PO_4_, as well as T and pH may be readily achieved by installation of simple sensors and basic testing protocols, the quantification of biomass of specific microbial groups and their corresponding intra-cellular fractions remains a challenge due to cost and/or complexity issues.

On the other hand, the uncertainty of experimental methods used to measure these quantities should be acknowledged and improved where possible. While quantification of the *Accumulibacter* PAO population in both laboratory and full-scale reactors using culture-independent molecular methods has become standard practice (López-Vázquez et al., 2007a; Oehmen et al., 2010b; Lanham et al., 2014), there is some controversy over the accuracy of such measurements.The relative abundances measured by different methods, e.g., quantitative fluorescence *in situ* hybridization (qFISH) via target sequences in the 16S rRNA molecule or real-time quantitative polymerase chain reaction (qPCR) via the poly-P kinase gene (*ppk1* are not always in complete agreement (Fukushima et al., 2007). This may be attributed to factors such as the sensitivity to low cell counts, differences in cellular metabolic activities, heterogeneities in experimental protocol (e.g., sample preparation), or sample characteristics (Moter and Göbel, 2000; van Loosdrecht et al., 2016; Huber et al., 2018). This is not to mention the added complication of the specificity of the technique to target the desired organism, e.g., the overlap of multiple FISH probes resulting in the over-estimation of *Accumulibacter* (Albertsen et al., 2016).

Similarly, while glycogen is an important source of carbon, energy (ATP) and reducing agents (NADH_2_) in both PAO and bacterial metabolisms in other environments, it has proven to be difficult to accurately quantify. Protocols depend on the type of cell and the state of cell-aggregation, i.e., on whether the biomass is floccular or granular (Lanham et al., 2012). Consequently, model predictions have been shown to exceed the measured concentrations of PHA and poly-P (Brdjanovic et al., 2000; Meijer et al., 2002). Lopez-Vazquez et al. (2009) found that current metabolic models consistently overestimated the *X*_Gly_ concentration by as much as 25%. In view of cumulative errors in long-term multi-cycle simulations, even small errors can lead to significant deviations in prediction accuracy. As such, the problem of accurate, fast and (if possible) simpler quantification of intra-cellular fractions, namely that of glycogen and PHA, requires more attention to reduce prediction error, and thus for the effective deployment of metabolic models in full-scale wastewater treatment plants.

#### 4.1.2. Influence of Temperature Coefficients

As shown in Figure 5 and Table 2, the Arrhenius temperature coefficients for anaerobic maintenance (θ_*m*_ATP, an__), aerobic glycogen production (θ_*q*_Gly__) and poly-P formation (θ_*q*_PP__) rank among the most influential inputs for all output variables. Although relatively abundant data is available for the kinetic uptake rates (Figure 1), relatively limited attention has been devoted to temperature coefficients, apart from the work of Brdjanovic et al. (1997, 1998). One limitation of this work was that the reported temperature dependencies have only been verified to a limited range of operating temperatures (typically between 10 and 20 ℃). Given that Arrhenius temperature coefficients are expected to take a constant value in theory, further investigation of the kinetic processes are required to specify the uncertainty in these parameters, and thereby ascertain the accuracy of model predictions.

This is of particular importance in the context of using metabolic models to study the competition between PAO and GAO, as performed by Lopez-Vazquez et al. (2009). It would also enable more accurate study of PAO kinetics at wider temperature ranges, particularly in light of recent, seemingly contradictory findings concerning the population dynamics of PAO and GAO at higher temperatures (Ong et al., 2014; Shen et al., 2017). Information concerning the temperature coefficients of (*Competibacter*) GAO mirror that of PAO, their values deriving from only two investigations (López-Vázquez et al., 2007b; Lopez-Vazquez et al., 2008a). Recent findings concerning the identity of *Accumulibacter* PAO clades, subdivisions of *Competibacter* GAO, as well as *Defluvicoccus*-related GAO (Oehmen et al., 2010b), call for a more comprehensive re-evaluation of the temperature dependencies to determine their “true” values.

#### 4.1.3. Influence of Kinetic Parameters

After considering initial conditions and temperature parameters, the burden of improving model reliability falls on reducing the input uncertainty associated with kinetic parameters. The priority should be the estimation of the maximum specific rates of anaerobic HPr uptake (*q*_HPr_), stand maintenance coefficient (*m*_ATP, an_), aerobic poly-P formation (*q*_PP_), glycogen production (*q*_Gly_) and the aerobic maintenance coefficient (*m*_ATP, ox_). Interestingly, these parameters were highly influential on the prediction of all model outputs, despite the fact that data concerning kinetic parameters were the most abundant. This was likely due to the comparatively large variance in the input values, notably in the case of *q*_PP_. This suggests that there may be a “natural” level of variation that can be expected in the values of certain kinetic parameters, notably in the maximum rates of change involved in Monod-type expressions, for which additional measurements will not lead to further improvements in model prediction accuracy. This inherent level of variation may be related to the characteristics of the biomass and/or influent of the system in question.

#### 4.1.4. Influence of Metabolic Parameters

As seen from Table 2, the most influential metabolic parameters were the half-saturation constants for anaerobic HPr uptake (*K*_HPr_), and PHA formation (*K*_PHA_), as well as those associated with aerobic glycogen (*K*_Gly_), poly-P formation (*K*_PP_) and δ. All of these are strongly linked to the transport of HPr across the cell membrane, storage of PHA and subsequent regeneration of glycogen. These parameters may become more influential in determining the composition of the PAO-GAO community in simulations involving both groups of microorganisms. As such, it is important that the uncertainty of these parameters be reduced to improve model prediction reliability, as the preferential uptake of HPr has been shown to be one of the key factors in the competition between the two groups of microorganisms (Oehmen et al., 2005b; Carvalheira et al., 2014b).

#### 4.1.5. Model Simplification by Fixing Non-influential Parameters

The contribution of input uncertainty on the variance of model predictions was found to vary from one output variable to another (Figure 5). For the prediction of liquid phase concentrations (*S*_O_2__, *S*_HAc_, *S*_HPr_, *S*_P_O__4__, *X*_PAO_), as well as intra-cellular concentrations of glycogen (*X*_Gly_) and poly-phosphate (*X*_PP_), the vast majority of uncertainty could be attributed to a smaller subset of input parameters (64%). This suggests that uncertainty in some input parameters did not affect the prediction accuracy of liquid phase components to an appreciable extent. These include the maximum specific rate of HAc uptake (*q*_PAO_) and aerobic PHA degradation (*q*_PHA_), the maximum intra-cellular fraction of poly-P (*f*_PP, max_), the aerobic maintenance coefficient (*m*_ATP, an_), the half-saturation constants for *f*_PHA_, PO_4_, O_2_ and HAc, as well as the phosphate transport coefficient (ε) and ATP yield per unit of biomass on Propionyl-CoA (*K*_2_). Concerning the prediction of intra-cellular PHA, the total-order sensitivity indices were significant and nearly uniformly distributed among all of these input parameters. Since uncertainty in these input parameters has little effect on the model output outside of the intra-cellular PHA concentrations, they could potentially be fixed to constant values.

Reducing the uncertainty regarding any of the aforementioned input parameters will significantly improve model reliability. In the context of simplifying metabolic models for integration within the wider ASM framework, these results merit further investigation to determine the trade-off between fixing the aforementioned input parameters, especially where the total-order index was lesser than 0.5, and the prediction error with regards to intra-cellular PHA.

### 4.2. Limitations and Future Perspectives

This work has for the first time assessed the overall effect of input parameter uncertainty on the variance of metabolic model predictions. Most of the propagated uncertainty propagation is not linear from one parameter to one output, but rather due to the interaction of multiple parameters. This brings conclusions in previous studies focused on parameter estimation for metabolic models in EBPR into question.Parameters such as the anaerobic maintenance coefficient (*m*_ATP, an_) and the yield of ATP per unit of NADH_2_ (δ) have often been justified as constants on the basis of local sensitivity analyses specific to particular conditions, typically in lab-scale reactors, e.g., in Zeng et al. (2003b).

This does not invalidate previous findings, as prediction uncertainty does indeed depend on the particular process conditions. However, in view of applying EBPR metabolic models to the general case, i.e., to characterize systems where the influent cannot be strictly controlled and/or process conditions vary spatially and temporally in the reactor, this study does highlight that local sensitivity analyses are not sufficient to identify regions in which current EBPR metabolic models may be accurately used. More data is needed to determine the true value or at the least the experimental range of a number of parameters. Baring that, if a natural degree of variation exists for certain input parameters, then its distribution should be defined.

Although the input space was sampled to conform to the assumptions of the metabolic model, i.e., within a narrow range of temperatures, pH and an influent mixture of HAc and HPr, the predictions may not be valid in situations where the PAO population is subject to limiting conditions for extended periods of time. For instance, PAO have been found to shift from PHA to glycogen accumulation in anaerobic conditions following a lack of PO_4_ in the influent (Acevedo et al., 2014, 2017)—a phenotypic behavior characteristic of their GAO competitors. Similarly, PAO have been shown to employ the tricarboxylic acid (TCA) cycle to supplement the production of reducing agents to cope with a lack of VFA in the influent, or due to glycogen depletion, possibly as a result of prolonged periods of cellular maintenance (Lanham et al., 2013, 2014). It has also been found that PAO require a longer period of adjustment compared to GAO following extended starvation periods (Vargas et al., 2013; Carvalheira et al., 2014c). As such, the model would require additional considerations of the kinetic and metabolic parameters, or (more likely) structural modifications to the formulation of kinetic rates in order to accurately reflect observations of such behavior in response to or during recovery from long-term duress.

This work used SRC and the Sobol method to conduct a global sensitivity analysis of a metabolic model for PAO. Various groups of input parameters were considered, including metabolic parameters, kinetic parameters, Arrhenius temperature coefficients and the initial conditions required to initialize the model. However, certain factors often held constant in laboratory-scale studies yet crucial to the operation of full-scale wastewater treatment plants were not accounted for. These included the sludge retention time (SRT), hydraulic retention time (HRT) and the lengths of the anaerobic and aerobic phases, which would often be the only means by which to adjust process conditions without interfering with the influent in the event of process disruption. Temperature and pH were also assumed constant over any given anaerobic-aerobic cycle.

Further, this work did not consider an anoxic phase, for which metabolic models have been formulated previously, e.g., in Oehmen et al. (2010b). This was for practical reasons, as the additional burden of quantifying the effect of uncertainty in parameters relevant to anoxic conditions would lead to an exponential increase in the number of model evaluations required to converge to the true value of (variance-based) sensitivity indices. Nevertheless, given the growing interest in anaerobic-aerobic-anoxic EBPR, owing to the potential of further energy savings from reduced aeration requirements, as well as simultaneous removal of nitrogen alongside phosphorus, future work may first consider the evaluation of parameter importance using computationally less expensive screening methods, e.g., the elementary effects (EE) method, where the convergence of sensitivity indices is less dependent of the number of model evaluations (Saltelli et al., 2008). In this way, non-influential parameters would be identified prior to more detailed investigations of the interactions between those input parameters which contribute most to the variance in model predictions. To account for interactions between different inputs, higher-order sensitivities should be accounted for. This is typically achieved by variance-based methods e.g., Sobol indices (Saltelli et al., 2008) and Mara's (2009) extention of RBD-FAST, or density-based methods such as those detailed in Plischke et al. (2013) and Pianosi and Wagener (2015).

Finally, the driving force for the development of metabolic models has been the prediction of conditions more favorable to PAO than GAO. As such, despite the absence of evidence suggesting co-metabolism, i.e., the lack of dependence of one organism on metabolites synthesized by the other, it would be worthwhile to complement the results obtained in this work with uncertainties in the prediction of concentrations for both *Competibacter* and *Defluvicoccus*-related GAO. This, as well as the differentiation between different PAO clades would merit further investigation to understand the uncertainty not only in the prediction of individual metabolic models, but also how it would propagate in studying the competition between organisms relevant to EBPR.

## 5. Conclusions

This work conducted an uncertainty and global sensitivity analysis of a metabolic model describing the behavior of PAO in alternating anaerobic and aerobic EBPR. The input uncertainty was characterized based on the relative abundance of data in the literature for each input parameter required to initialize the model. The input uncertainty was propagated to the output using the Monte Carlo method. Differences in the variance of the mean concentration profiles indicated that concentrations of phosphate in the bulk liquid phase and biomass were the most uncertain, whereas the concentrations of intra-cellular PHV, poly-P and PH_2_MV were the least uncertain.

The global sensitivity analysis was conducted using SRC and Sobol sensitivity indices. The analysis comprised a total of 39 input parameters: 12 metabolic parameters, 10 kinetic parameters, 6 Arrhenius temperature coefficients and 9 initial conditions (influent), including temperature and pH. SRC were found to be an inadequate sensitivity measure for this model due to the low degree of linearity between the input parameters and the output variables. Consequently, reduction of model complexity by linearization of its parts is not feasible in the general case, and limited to particular subsets of output variables for more specific scenarios with well-defined initial conditions. Differences between first and total-order Sobol indices indicated that the variance in model predictions was mainly due to interaction effects between combinations of input parameters rather than the uncertainty of individual ones.

The contribution of each of the input parameters on the uncertainty of model predictions varied with the output variable in question. For the prediction of liquid phase concentrations and intra-cellular fractions of glycogen and poly-phosphate, the vast majority of the uncertainty could be attributed to a smaller subset of input parameters (64% ). For the prediction of intra-cellular PHA constituents, the contribution was nearly uniformly distributed among all input parameters, indicating a high-degree of interaction. Although the contribution could not be isolated to any particular group of inputs (metabolic parameters, kinetic parameters, temperature coefficients, process conditions), the initial fractions of PHV, PH_2_MV and glycogen ranked consistently among the most influential factors, both in terms of direct as well as total effect, suggesting that the value of these parameters should be carefully measured when applying EBPR metabolic models.

This work contributed a step toward a more complete understanding of the uncertainties associated with EBPR metabolic model predictions, and how to address these uncertainties on an individual basis given knowledge of the corresponding input uncertainty. Possible approaches and pre-requisite conditions to simplify metabolic models for PAO, both structurally via linearization, as well as by reducing the number of non-influential variables were illustrated based on the results of the sensitivity analyses. Parameters requiring further experimental consideration were highlighted. This will translate to more accurate prediction of PAO behavior, thus facilitating process monitoring and control. Further, the findings of this work will lead to more informed decision-making in model building and in fundamental investigations of organisms relevant to EBPR systems.

## Author Contributions

MN formulated and implemented the model, performed the numerical simulations, analyzed the data, and drafted the manuscript. AL and TR contributed to experimental design. All authors contributed to manuscript revision, read, and approved the submitted version.

### Conflict of Interest

The authors declare that the research was conducted in the absence of any commercial or financial relationships that could be construed as a potential conflict of interest.

## Figures and Tables

**Table:** 

**Symbol**	**Meaning**	**Units**
EBPR	enhanced biological phosphorus removal	
AS	activated sludge	
ASM	activated sludge model	
SRC	standard regression coefficients	
OLS	ordinary least squares	
LASSO	least absolute shrinkage and selection operator	
RBD-FAST	Fourier amplitude sensitivity test via random balanced designs	
GSA	global sensitivity analysis	
PHA	poly-β-hydroxy-alkanoate	
PHB	poly-β-hydroxy-butyrate	
PHV	poly-β-hydroxy-valerate	
PH_2_MV	poly-β-hydroxy-methyvalerate	
PAO	phosphorus accumulating organism	
GAO	glycogen accumulating organism	
poly-P	poly-phosphate	
VFA	volatile fatty acids	
HAc	acetate	
HPr	propionate	
SRT	solids retention time	
HRT	hydraulic retention time	
T	temperature	°C
*X*_j_	intra-cellular concentration of component *j*	C-mol/l
*X*_j, i_	initial intra-cellular concentration of component *j*	P or C-mol/l
*f*_j_	intra-cellular fraction of component *j* per unit of biomass	P or C-mol/C-mol
*f*_j*i*_	initial intra-cellular fraction of component *j* per unit of biomass	P or C-mol/C-mol
*f*j, max	maximum intra-cellular fraction of component *j* per unit of biomass	P or C-mol/C-mol
*S*_j_	concentration of *j* in the bulk liquid phase	P or C-mol/l
*S*_VFA, i_	initial concentration of *j* in the bulk liquid phase	C-mol/l
*r*_P/C, i_	ratio of the PO_4_ to VFA concentration in the influent	
*r*_HAc/HPr, i_	ratio of acetate to propionate in the influent VFA	
*q*_j_	maximum rate of conversion of component *j*	P or C-mol/C-molh^−1^
*m*_ATP, an_	anaerobic maintenance coefficient	P-mol/C-molh^−1^
*m*_ATP, ox_	aerobic maintenance coefficient	ATP-mol/C-molh^−1^
*K*_j_	half-saturation constant of component *j*	C-mol/l
θ_j_	Arrhenius temperature coefficient of component *j*	
δ	yield of ATP per unit of NADH oxidized (P/O ratio)	ATP-mol/NADH-mol
ε	aerobic PO_4_ transport coefficient	P-mol/NADH-mol
*K*_1_	ATP required for biomass synthesis from Acetyl-CoA*	ATP-mol/C-mol
*K*_2_	ATP required for biomass synthesis from Propionyl-CoA*	ATP-mol/C-mol
