# Supplementary material for: Global Sensitivity Analysis of Metabolic Models for Phosphorus Accumulating Organisms in Enhanced Biological Phosphorus Removal
Source: Front Bioeng Biotechnol. 2019 Oct 4;7:234. doi: 10.3389/fbioe.2019.00234 (PMC6787149; doi:10.3389/fbioe.2019.00234)
Supplement: Supplementary file 1 [file Data_Sheet_1.PDF]

# Supplementary Material

## Global Sensitivity Analysis of Metabolic Models for Phosphorus Accumulating Organisms in Enhanced Biological Phosphorus Removal

Minh Nguyen Quang [1], Tim Rogers [2], Jan Hofman [1] and Ana B Lanham [1,*]

[1] Water Innovation and Research Centre, Department of Chemical Engineering, University of Bath, Bath, United Kingdom
[2] Centre for Networks and Collective Behaviour, Department of Mathematical Sciences, University of Bath, Bath, United Kingdom

* Corresponding author: a.lanham@bath.ac.uk

Index of supplementary materials

| Item | Description |
|---|---|
| Appendix 1 | Stoichiometric matrix |
| Appendix 2 | Anaerobic stoichiometric coefficients |
| Appendix 3 | Aerobic stoichiometric coefficients |
| Appendix 4 | Kinetic parameters |
| Appendix 5 | Kinetic expressions |
| Appendix 6 | Input parameter values reported in the literature |
| Appendix 7 | Input data samples for Monte Carlo simulations |
| Appendix 8 | Standard Regression coefficients, including $R^2$ values corresponding to each output |
| Appendix 9 | First-order Sobol sensitivity indices |
| Appendix 10 | Confidence intervals corresponding to the first-order Sobol sensitivities (Appendix 9) |
| Appendix 11 | Total-order Sobol sensitivity indices |
| Appendix 12 | Confidence intervals corresponding to the total-order Sobol sensitivities (Appendix 11) |
| Appendix 13 | Mean of the concentration profiles obtained by Monte Carlo simulations |

Appendices 6-13 are provided as .csv files.

**Appendix 1:** Stoichiometric matrix

| Process | | Component | | | | | | | | | |
|---|---|---|---|---|---|---|---|---|---|---|---|
| | | $S_{O_2}$ | $S_{HAc}$ | $S_{HPr}$ | $S_{PO_4}$ | $X_{PAO}$ | $X_{PHB,PAO}$ | $X_{PHV,PAO}$ | $X_{PH_2MV,PAO}$ | $X_{Gly,PAO}$ | $X_{PP,PAO}$ |
| Anaerobic | HAc uptake | | $-1$ | | $Y_{PO_4,HAc}$ | | $Y_{PHB,HAc}$ | $Y_{PHV,HAc}$ | $Y_{PH_2MV,HAc}$ | $-Y_{Gly,HAc}$ | $-Y_{PP,HAc}$ |
| | HPr uptake | | | $-1$ | $Y_{PO_4,HPr}$ | | $Y_{PHB,HPr}$ | $Y_{PHV,HPr}$ | $Y_{PH_2MV,HPr}$ | $-Y_{Gly,HPr}$ | $-Y_{PP,HPr}$ |
| | Maintenance on poly-P | | | | $1$ | | | | | | $-1$ |
| | Maintenance on glycogen | | | | | | $1/6$ | $5/12$ | $1/4$ | $-1$ | |
| Aerobic | PHB degradation | $-Y_{PHA,O_2}$ | | | $-Y_{PO_4,X}$ | $Y_{PHA,X}$ | $-1$ | | | | |
| | PHV degradation | $-Y_{PHA,O_2}$ | | | $-Y_{PO_4,X}$ | $Y_{PHA,X}$ | | $-1$ | | | |
| | $PH_2MV$ degradation | $-Y_{PHA,O_2}$ | | | $-Y_{PO_4,X}$ | $Y_{PHA,X}$ | | | $-1$ | | |
| | Glycogen production | $Y_{Gly,O_2}$ | | | $Y_{PO_4,Gly}$ | $-Y_{X,Gly}$ | | | | $1$ | |
| | Poly-P formation | $Y_{PP,O_2}$ | | | $-Y_{PO_4,PP}$ | $-Y_{X,PP}$ | | | | | $1$ |
| | Maintenance on PHA | | $\frac{Y_{PHA,X}}{Y_{O_2,X}} - 1$ | | $i_P\,Y_{PHA,X}$ | $-Y_{PHA,X}$ | $-Y_{PHA,X}$ | $-Y_{PHA,X}$ | $-Y_{PHA,X}$ | | |
| | Maintenance on glycogen | | $-1/4$ | | | | $2/3$ | | | $-1$ | |
| | Maintenance on poly-P | | | | $1$ | | | | | | $-1$ |

**Appendix 2:** Anaerobic stoichiometric coefficients for PAO (Smolders et al., 1994a; Filipe et al., 2001b; Oehmen et al., 2005a,b)

| Parameter | Value | Units |
|---|---|---|
| $Y_{\mathrm{PHB,HAc}}$ | $\frac{4}{3}X_{\mathrm{PHB}}$ | C-mmol/C-mmol |
| $Y_{\mathrm{PHV,HAc}}$ | 0 | C-mmol/C-mmol |
| $Y_{\mathrm{PH_2MV,HAc}}$ | 0 | C-mmol/C-mmol |
| $Y_{\mathrm{PO_4,HAc}}$ | $0.5+\alpha_{\mathrm{HAc}}$ | P-mmol/C-mmol |
| $Y_{\mathrm{Gly,HAc}}$ | 0.5 | C-mmol/C-mmol |
| $Y_{\mathrm{PHB,HPr}}$ | 0 | C-mmol/C-mmol |
| $Y_{\mathrm{PHV,HPr}}$ | $0.56$ if $pH \leq 7$ else $(2.022-0.21pH)X_{\mathrm{PHV}}$ | C-mmol/C-mmol |
| $Y_{\mathrm{PH_2MV,HPr}}$ | $0.67$ if $pH \leq 7$ else $(1.172-0.07pH)X_{\mathrm{PH_2MV}}$ | C-mmol/C-mmol |
| $Y_{\mathrm{PO_4,HPr}}$ | $0.3$ if $pH \leq 7.5$ else $0.2pH-1.2$ | P-mmol/C-mmol |
| $Y_{\mathrm{Gly,HPr}}$ | $\frac{1}{3}$ if $pH \leq 7$ else $1.73-0.2pH$ | C-mmol/C-mmol |
| $\alpha_{\mathrm{HAc}}$ | $0.16pH-0.7985$ | ATP-mmol/C-mmol |

**Appendix 3:** Aerobic stoichiometric coefficients for PAO
(Smolders et al., 1994b; Zeng et al., 2003b; Lopez-Vazquez et al., 2009)

| Parameter | Value | Units | Description |
|---|---|---|---|
| $i_{\mathrm{H}}$ | 1.84 | H-mmol/C-mmol | Biomass hydroden content |
| $i_{\mathrm{O}}$ | 0.5 | O-mmol/C-mmol | Biomass oxygen content |
| $i_{\mathrm{N}}$ | 0.19 | N-mmol/C-mmol | Biomass nitrogen content |
| $i_{\mathrm{P}}$ | 0.015 | P-mmol/C-mmol | Biomass phosphorus content |
| $Redox_{\mathrm{X}}$ | $4+i_{\mathrm{H}}+5i_{\mathrm{P}}-2i_{\mathrm{O}}-3i_N$ | $e^-$ per C-mmol | Degree of biomass reduction |
| $Redox_{\mathrm{PHA}}$ | $\frac{4.5X_{\mathrm{PHB}}+4.8X_{\mathrm{PHV}}+5X_{\mathrm{PH_2MV}}}{X_{\mathrm{PHA}}}$ | $e^-$ per C-mmol | Degree of PHA reduction |
| $\lambda$ | $\frac{X_{\mathrm{PHB}}+\frac{2}{5}X_{\mathrm{PHV}}}{X_{\mathrm{PHA}}}$ | C-mmol/C-mmol | Fraction of Acetyl-CoA in PHA |
| $\beta$ | $\frac{X_{\mathrm{PH_2MV}}+\frac{3}{5}X_{\mathrm{PHV}}}{X_{\mathrm{PHA}}}$ | C-mmol/C-mmol | Fraction Propionyl-CoA in PHA |
| $Y_{\mathrm{PHA,X}}$ | $\frac{250(106\lambda+127\beta)(6\lambda+27\lambda\beta+8\beta+30\beta\delta)}{201930\lambda+318000K_1\lambda+678771\lambda\delta+269240\beta+381000K_2\beta+813435\beta\delta)}$ | C-mmol/C-mmol | Max yield of biomass growth on PHA |
| $Y_{\mathrm{PHA,Gly}}$ | $\frac{(3\lambda+4\beta)(6\lambda+27\lambda\beta+8\beta+30\beta\delta)}{24(2\lambda+3\lambda\beta+2\beta+4\beta\delta)}$ | C-mmol/C-mmol | Max yield of glycogen stored on PHA |
| $Y_{\mathrm{PHA,PP}}$ | $\frac{\varepsilon(6\lambda+27\lambda\beta+8\beta+30\beta\delta)}{12(\varepsilon+\delta)}$ | P-mmol/C-mmol | Max yield of poly-P stored on PHA |
| $Y_{\mathrm{O_2,X}}$ | $\left(\frac{Redox_{\mathrm{PHA}}}{4Y_{\mathrm{PHA,X}}}-\frac{Redox_{\mathrm{X}}}{4}\right)^{-1}$ | C-mmol/$O_2$-mmol | |

**Appendix 3:** Aerobic stoichiometric coefficients for PAO
(Smolders et al., 1994b; Zeng et al., 2003b; Lopez-Vazquez et al., 2009)

| Parameter | Value | Units |
|---|---|---|
| $Y_{O_2,Gly}$ | $\left(\frac{Redox_{PHA}}{4Y_{PHA,Gly}}-1\right)^{-1}$ | C-mmol/$O_2$-mmol |
| $Y_{O_2,PP}$ | $\left(\frac{Redox_{PHA}}{4Y_{PHA,PP}}\right)^{-1}$ | P-mmol/$O_2$-mmol |
| $Y_{PHA,O_2}$ | $\frac{Y_{PHA,X}}{Y_{O_2,X}}$ | $O_2$-mmol/C-mmol |
| $Y_{Gly,O_2}$ | $\frac{Y_{PHA,X}}{Y_{O_2,X}Y_{PHA,Gly}}-\frac{1}{Y_{O_2,Gly}}$ | $O_2$-mmol/C-mmol |
| $Y_{PP,O_2}$ | $\frac{Y_{PHA,X}}{Y_{O_2,X}Y_{PHA,PP}}-\frac{1}{Y_{O_2,PP}}$ | $O_2$-mmol/P-mmol |
| $Y_{PO_4,X}$ | $i_P Y_{PHA,X}$ | P-mmol/C-mmol |
| $Y_{PO_4,Gly}$ | $i_P\frac{Y_{PHA,X}}{Y_{PHA,Gly}}$ | P-mmol/C-mmol |
| $Y_{PO_4,PP}$ | $i_P\frac{Y_{PHA,X}}{Y_{PHA,PP}}-1$ | P-mmol/P-mmol |
| $Y_{X,Gly}$ | $\frac{Y_{PHA,X}}{Y_{PHA,Gly}}$ | C-mmol/C-mmol |
| $Y_{X,PP}$ | $\frac{Y_{PHA,X}}{Y_{PHA,PP}}$ | C-mmol/C-mmol |

**Appendix 4:** Kinetic parameters
(Brdjanovic et al., 1998; Lopez-Vazquez et al., 2009; Lanham et al., 2014; Carvalheira et al., 2014b)

| Parameter | Value | Units | Description |
|---|---|---|---|
| $m_{\mathrm{an}}$ | $m_{\mathrm{ATP,an}}\,\theta_{m_{\mathrm{ATP,an}}}{}^{(T-20)}$ | C-mol/C-molh$^{-1}$ | Anaerobic maintenance coefficient |
| $m_{\mathrm{PHA}}$ | $\frac{12m_{\mathrm{ATP,ox}}}{6+27\delta}\,\theta_{q_{\mathrm{VFA}}}{}^{(T-20)}$ | C-mol/C-molh$^{-1}$ | Aerobic maintenance coefficient on PHA |
| $m_{\mathrm{Gly}}$ | $\frac{2m_{\mathrm{ATP,ox}}}{1+\delta}\,\theta_{q_{\mathrm{VFA}}}{}^{(\mathrm{T}-20)}$ | C-mol/C-molh$^{-1}$ | Aerobic maintenance coefficient on glycogen |
| $m_{\mathrm{PP}}$ | $m_{\mathrm{ATP,ox}}\,\theta_{q_{\mathrm{VFA}}}{}^{(T-20)}$ | P-mol/C-molh$^{-1}$ | Aerobic maintenance coefficient on poly-P |
| $q_{\mathrm{HAc}}$ | $q_{\mathrm{VFA,max}}-q_{\mathrm{HPr,max}}\quad \text{if } S_{\mathrm{HPr}}>K_{\mathrm{HPr}}\quad \text{else}$ | C-mol/C-molh$^{-1}$ | Specific rate of anaerobic acetate uptake |
| | $q_{\mathrm{HAc,max}}\left(\theta_{q_{\mathrm{VFA}}}{}^{(T-20)}\quad \text{if } T\leq 20\right)$ | | |
| $q_{\mathrm{HPr}}$ | $q_{\mathrm{HPr,max}}\left(\theta_{q_{\mathrm{VFA}}}{}^{(T-20)}\quad \text{if } T\leq 20\right)$ | C-mol/C-molh$^{-1}$ | Specific rate of anaerobic HPr uptake |
| $q_{\mathrm{PHA}}$ | $q_{\mathrm{PHA,max}}\left(\theta_{q_{\mathrm{PHA}}}{}^{(T-20)}\quad \text{if } T\leq 20\right)$ | C-mol/C-molh$^{-1}$ | Specific rate of aerobic PHA degradation |
| $q_{\mathrm{Gly}}$ | $q_{\mathrm{Gly,max}}\left(\theta_{q_{\mathrm{Gly}}}{}^{(T-20)}\quad \text{if } T\leq 20\right)$ | C-mol/C-molh$^{-1}$ | Specific rate of aerobic glycogen production |
| $q_{\mathrm{PP}}$ | $q_{\mathrm{PP,max}}\left(\theta_{q_{\mathrm{PP}}}{}^{(T-20)}\quad \text{if } T\leq 20\right)$ | P-mol/C-molh$^{-1}$ | Specific rate of aerobic poly-P formation |

**Appendix 5:** Kinetic expressions
(Murnleitner et al., 1997; Meijer et al., 2002; Lopez-Vazquez et al., 2009; Oehmen et al., 2010; Lanham et al., 2014)

| Conditions | Process | Expression | Switch function |
|---|---|---|---|
| Anaerobic | HAc uptake | $q_{\mathrm{HAc}} \frac{S_{\mathrm{HAc}}}{S_{\mathrm{HAc}} + K_{\mathrm{HAc}}}$ | $\frac{X_{\mathrm{PP}}}{X_{\mathrm{PP}}} + K_{\mathrm{PP}} \frac{X_{\mathrm{Gly}}}{X_{\mathrm{Gly}} + K_{\mathrm{Gly}}} \frac{f_{\mathrm{PHA,\,max}} - f_{\mathrm{PHA}}}{f_{\mathrm{PHA,\,max}} - f_{\mathrm{PHA}} + K_{\mathrm{PHA}}} \left(1 - \frac{S_{\mathrm{O_2}}}{S_{\mathrm{O_2}} + K_{\mathrm{O_2}}}\right)$ |
| | HPr uptake | $q_{\mathrm{HPr}} \frac{S_{\mathrm{HPr}}}{S_{\mathrm{HPr}} + K_{\mathrm{HPr}}}$ | $\frac{X_{\mathrm{PP}}}{X_{\mathrm{PP}} + K_{\mathrm{PP}}} \frac{X_{\mathrm{Gly}}}{X_{\mathrm{Gly}} + K_{\mathrm{Gly}}} \frac{f_{\mathrm{PHA,\,max}} - f_{\mathrm{PHA}}}{f_{\mathrm{PHA,\,max}} - f_{\mathrm{PHA}} + K_{\mathrm{PHA}}} \left(1 - \frac{S_{\mathrm{O_2}}}{S_{\mathrm{O_2}} + K_{\mathrm{O_2}}}\right)$ |
| | Maintenance on poly-P | $m_{\mathrm{ATP,\,an}}$ | $\frac{X_{\mathrm{PP}}}{X_{\mathrm{PP}} + K_{\mathrm{PP}}} \left(1 - \frac{S_{\mathrm{O_2}}}{S_{\mathrm{O_2}} + K_{\mathrm{O_2}}}\right)$ |
| | Maintenance on glycogen | $m_{\mathrm{ATP,\,an}}$ | $\frac{X_{\mathrm{Gly}}}{X_{\mathrm{Gly}} + K_{\mathrm{Gly}}} \left(1 - \frac{X_{\mathrm{PP}}}{X_{\mathrm{PP}} + K_{\mathrm{PP}}}\right) \left(1 - \frac{S_{\mathrm{O_2}}}{S_{\mathrm{O_2}} + K_{\mathrm{O_2}}}\right)$ |
| Aerobic | PHA degradation | $q_{\mathrm{PHA}}\, f_{\mathrm{PHA}}{}^{2/3}$ | $\frac{X_{\mathrm{PHA}}}{X_{\mathrm{PHA}} + K_{\mathrm{PHA}}} \frac{S_{\mathrm{O_2}}}{S_{\mathrm{O_2}} + K_{\mathrm{O_2}}}$ |
| | Glycogen production | $q_{\mathrm{Gly}}\, f_{\mathrm{PHA}}{}^{2/3}\, f_{\mathrm{Gly}}{}^{-1}$ | $\frac{f_{\mathrm{Gly,\,max}} - f_{\mathrm{Gly}}}{f_{\mathrm{Gly,\,max}} - f_{\mathrm{Gly}} + K_{\mathrm{Gly}}} \frac{X_{\mathrm{PHA}}}{X_{\mathrm{PHA}} + K_{\mathrm{PHA}}} \frac{S_{\mathrm{O_2}}}{S_{\mathrm{O_2}} + K_{\mathrm{O_2}}}$ |
| | Poly-P formation | $q_{\mathrm{PP}}\, f_{\mathrm{PP}}{}^{-1}$ | $\frac{f_{\mathrm{PP,\,max}} - f_{\mathrm{PP}}}{f_{\mathrm{PP,\,max}} - f_{\mathrm{PP}} + K_{\mathrm{PP}}} \frac{X_{\mathrm{PHA}}}{X_{\mathrm{PHA}} + K_{\mathrm{PHA}}} \frac{S_{\mathrm{PO_4}}}{S_{\mathrm{PO_4}} + K_{\mathrm{PO_4}}} \frac{S_{\mathrm{O_2}}}{S_{\mathrm{O_2}} + K_{\mathrm{O_2}}}$ |
| | Maintenance on PHA | $m_{\mathrm{PHA}}$ | $\frac{X_{\mathrm{PHA}}}{X_{\mathrm{PHA}} + K_{\mathrm{PHA}}} \frac{S_{\mathrm{O_2}}}{S_{\mathrm{O_2}} + K_{\mathrm{O_2}}}$ |
| | Maintenance on glycogen | $m_{\mathrm{Gly}}$ | $\frac{X_{\mathrm{Gly}}}{X_{\mathrm{Gly}} + K_{\mathrm{Gly}}} \left(1 - \frac{X_{\mathrm{PHA}}}{X_{\mathrm{PHA}} + K_{\mathrm{PHA}}}\right) \frac{S_{\mathrm{O_2}}}{S_{\mathrm{O_2}} + K_{\mathrm{O_2}}}$ |
| | Maintenance on poly-P | $m_{\mathrm{PP}}$ | $\frac{X_{\mathrm{PP}}}{X_{\mathrm{PP}} + K_{\mathrm{PP}}} \left(1 - \frac{X_{\mathrm{PHA}}}{X_{\mathrm{PHA}} + K_{\mathrm{PHA}}}\right) \frac{S_{\mathrm{O_2}}}{S_{\mathrm{O_2}} + K_{\mathrm{O_2}}} (1 - \frac{X_{\mathrm{Gly}}}{X_{\mathrm{Gly}} + K_{\mathrm{Gly}}}) \left(1 - \frac{X_{\mathrm{PHA}}}{X_{\mathrm{PHA}} + K_{\mathrm{PHA}}}\right) \frac{S_{\mathrm{O_2}}}{S_{\mathrm{O_2}} + K_{\mathrm{O_2}}}$ |

## References


Acevedo, B., Borras, L., Oehmen, A., and Barat, R. (2014). Modelling the metabolic shift of polyphosphate-accumulating organisms. *Water Research* 65, 235–244

Brdjanovic, D., Logemann, S., van Loosdrecht, M. C., Hooijmans, C. M., Alaerts, G. J., and Heijnen, J. J. (1998). Influence of temperature on biological phosphorus removal: process and molecular ecological studies. *Water Research* 32, 1035–1048

Brdjanovic, D., Loosdrecht, M. C. v., Hooijmans, C. M., Alaerts, G. J., and Heijnen, J. J. (1997). Temperature effects on physiology of biological phosphorus removal. *Journal of Environmental Engineering* 123, 144–153

Carvalheira, M., Oehmen, A., Carvalho, G., Eusébio, M., and Reis, M. A. (2014a). The impact of aeration on the competition between polyphosphate accumulating organisms and glycogen accumulating organisms. *Water Research* 66, 296–307

Carvalheira, M., Oehmen, A., Carvalho, G., and Reis, M. A. (2014b). The effect of substrate competition on the metabolism of polyphosphate accumulating organisms (paos). *Water Research* 64, 149–159

Carvalheira, M., Oehmen, A., Carvalho, G., and Reis, M. A. (2014c). Survival strategies of polyphosphate accumulating organisms and glycogen accumulating organisms under conditions of low organic loading. *Bioresource Technology* 172, 290–296

Filipe, C. D., Daigger, G. T., and Grady, C. (2001a). Effects of ph on the rates of aerobic metabolism of phosphate-accumulating and glycogen-accumulating organisms. *Water Environment Research* 73, 213–222

Filipe, C. D., Daigger, G. T., and Grady Jr, C. L. (2001b). Stoichiometry and kinetics of acetate uptake under anaerobic conditions by an enriched culture of phosphorus-accumulating organisms at different phs. *Biotechnology and Bioengineering* 76, 32–43

Kuba, T., Murnleitner, E., Van Loosdrecht, M., and Heijnen, J. (1996). A metabolic model for biological phosphorus removal by denitrifying organisms. *Biotechnology and Bioengineering* 52, 685–695

Kuba, T., Smolders, G., Van Loosdrecht, M., and Heijnen, J. (1993). Biological phosphorus removal from wastewater by anaerobic-anoxic sequencing batch reactor. *Water Science and Technology* 27, 241–252

Lanham, A., Moita, R., Lemos, P., and Reis, M. (2011). Long-term operation of a reactor enriched in accumulibacter clade i dpaos: performance with nitrate, nitrite and oxygen. *Water Science and Technology* 63, 352–359

Lanham, A. B., Oehmen, A., Saunders, A. M., Carvalho, G., Nielsen, P. H., and Reis, M. A. (2014). Metabolic modelling of full-scale enhanced biological phosphorus removal sludge. *Water Research* 66, 283–295

Lopez-Vazquez, C. M., Oehmen, A., Hooijmans, C. M., Brdjanovic, D., Gijzen, H. J., Yuan, Z., et al. (2009). Modeling the pao–gao competition: effects of carbon source, ph and temperature. *Water Research* 43, 450–462

Meijer, S., Van Loosdrecht, M., and Heijnen, J. (2002). Modelling the start-up of a full-scale biological phosphorous and nitrogen removing wwtp. *Water Research* 36, 4667–4682

Murnleitner, E., Kuba, T., Van Loosdrecht, M., and Heijnen, J. (1997). An integrated metabolic model for the aerobic and denitrifying biological phosphorus removal. *Biotechnology and Bioengineering* 54, 434–450

Oehmen, A., Lopez-Vazquez, C., Carvalho, G., Reis, M., and Van Loosdrecht, M. (2010). Modelling the population dynamics and metabolic diversity of organisms relevant in anaerobic/anoxic/aerobic enhanced biological phosphorus removal processes. *Water Research* 44, 4473–4486

Oehmen, A., Vives, M. T., Lu, H., Yuan, Z., and Keller, J. (2005a). The effect of ph on the competition between polyphosphate-accumulating organisms and glycogen-accumulating organisms. *Water Research* 39, 3727–3737

Oehmen, A., Zeng, R. J., Keller, J., and Yuan, Z. (2007). Modeling the aerobic metabolism of polyphosphate-accumulating organisms enriched with propionate as a carbon source. *Water Environment Research* 79, 2477–2486

Oehmen, A., Zeng, R. J., Yuan, Z., and Keller, J. (2005b). Anaerobic metabolism of propionate by polyphosphate-accumulating organisms in enhanced biological phosphorus removal systems. *Biotechnology and Bioengineering* 91, 43–53

Pijuan, M., Oehmen, A., Baeza, J. A., Casas, C., and Yuan, Z. (2008). Characterizing the biochemical activity of full-scale enhanced biological phosphorus removal systems: a comparison with metabolic models. *Biotechnology and Bioengineering* 99, 170–179

Pijuan, M., Saunders, A. M., Guisasola, A., Baeza, J., Casas, C., and Blackall, L. (2004). Enhanced biological phosphorus removal in a sequencing batch reactor using propionate as the sole carbon source. *Biotechnology and Bioengineering* 85, 56–67

Rubio-Rincón, F., Lopez-Vazquez, C., Welles, L., van Loosdrecht, M., and Brdjanovic, D. (2017). Cooperation between candidatus competibacter and candidatus accumulibacter clade i, in denitrification and phosphate removal processes. *Water Research* 120, 156–164

Saito, T., Brdjanovic, D. v., and Van Loosdrecht, M. (2004). Effect of nitrite on phosphate uptake by phosphate accumulating organisms. *Water Research* 38, 3760–3768

Shen, N. and Zhou, Y. (2016). Enhanced biological phosphorus removal with different carbon sources. *Applied Microbiology and Biotechnology* 100, 4735–4745

Smolders, G., Van der Meij, J., Van Loosdrecht, M., and Heijnen, J. (1994a). Model of the anaerobic metabolism of the biological phosphorus removal process: stoichiometry and ph influence. *Biotechnology and Bioengineering* 43, 461–470

Smolders, G., Van der Meij, J., Van Loosdrecht, M., and Heijnen, J. (1994b). Stoichiometric model of the aerobic metabolism of the biological phosphorus removal process. *Biotechnology and Bioengineering* 44, 837–848

Smolders, G., Van der Meij, J., Van Loosdrecht, M., and Heijnen, J. (1995). A structured metabolic model for anaerobic and aerobic stoichiometry and kinetics of the biological phosphorus removal process. *Biotechnology and Bioengineering* 47, 277–287

Sudiana, I., Mino, T., Satoh, H., Nakamura, K., and Matsuo, T. (1999). Metabolism of enhanced biological phosphorus removal and non-enhanced biological phosphorus removal sludge with acetate and glucose as carbon source. *Water Science and Technology* 39, 29–35

Vargas, M., Guisasola, A., Artigues, A., Casas, C., and Baeza, J. (2011). Comparison of a nitrite-based anaerobic–anoxic ebpr system with propionate or acetate as electron donors. *Process Biochemistry* 46, 714–720

Vargas, M., Yuan, Z., and Pijuan, M. (2013). Effect of long-term starvation conditions on polyphosphate-and glycogen-accumulating organisms. *Bioresource Technology* 127, 126–131

Welles, L., Tian, W., Saad, S., Abbas, B., Lopez-Vazquez, C., Hooijmans, C., et al. (2015). Accumulibacter clades type i and ii performing kinetically different glycogen-accumulating organisms metabolisms for anaerobic substrate uptake. *Water Research* 83, 354–366

Zeng, R. J., Saunders, A. M., Yuan, Z., Blackall, L. L., and Keller, J. (2003a). Identification and comparison of aerobic and denitrifying polyphosphate-accumulating organisms. *Biotechnology and Bioengineering* 83, 140–148

Zeng, R. J., Van Loosdrecht, M. C., Yuan, Z., and Keller, J. (2003b). Metabolic model for glycogen-accumulating organisms in anaerobic/aerobic activated sludge systems. *Biotechnology and Bioengineering* 81, 92–105

Zhou, Y., Pijuan, M., Zeng, R. J., Lu, H., and Yuan, Z. (2008). Could polyphosphate-accumulating organisms (paos) be glycogen-accumulating organisms (gaos)? *Water Research* 42, 2361–2368
